# ﻿ *Coleus* (Lamiaceae) in Peninsular Malaysia including two new species

**DOI:** 10.3897/phytokeys.186.62018

**Published:** 2021-12-06

**Authors:** Ruth Kiew, Imin Kamin

**Affiliations:** 1 Forest Research Institute Malaysia, 52109 Kepong, Selangor, Malaysia Forest Research Institute Malaysia Kepong Malaysia

**Keywords:** Conservation status, limestone hills, *Plectranthus*, *Solenostemonmonostachyus*, *Solenostemonscutellarioides*

## Abstract

In Peninsular Malaysia, *Coleus* is represented by five species. Two, *C.hairulii* Kiew and *C.rafidahiae* Kiew, are new species. Both are narrowly endemic and restricted to limestone hills as is *C.kunstleri* (Prain) A.J.Paton. All three are Critically Endangered. *Coleusscutellarioides* (L.) Benth., although widespread, is probably not indigenous. It is also a common ornamental, while *C.monostachyus* (P.Beauv.) A.J.Paton is a recent introduction that has spread rapidly and threatens to become a troublesome weed.

## ﻿Introduction

**Map 1.**
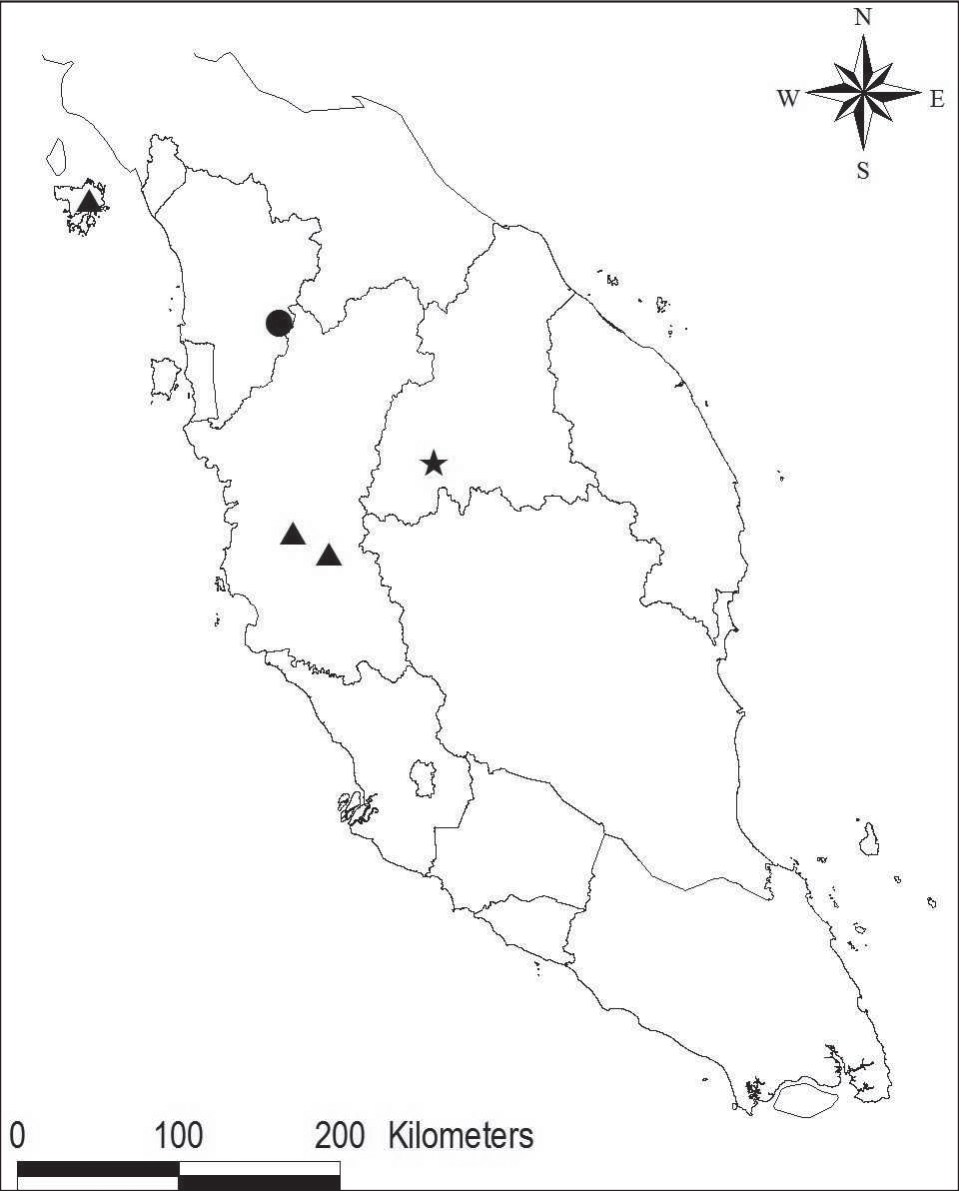
Distribution of *Coleushairulii* (circle), *C.kunstleri* (triangle) and *C.rafidahiae* (star) in Peninsular Malaysia.

The flora of Peninsular Malaysia now includes five species of *Coleus*, compared with the two previously recorded under *Plectranthus* by Keng (1978) and Bramley (2019). *Coleus* has recently been recognised as distinct from *Plectranthus* (Paton et al. 2019). *Coleusscutellarioides* (L.) Benth. is a widespread lowland species, often associated with villages, and *C.kunstleri* (Prain) A.J. Paton is very rare and known from just two limestone localities. Recent exploration of limestone karsts in Peninsular Malaysia has led to the discovery of the two new species that are described here. *Coleusmonostachyus* (P.Beauv.) A.J. Paton is an African weed recently introduced and now, after a decade, has rapidly become widespread and is found everywhere in ruderal habitats (Kiew 2016). The three limestone species are endemic and restricted to karst limestone (Map 1).

## ﻿Conservation

Most of the 445 or so limestone karst hills or islands in Peninsular Malaysia are small with a basal area of about 1 km^2^ (Liew et al. 2016). Together they cover about 0.2% of land area but support about 14% of the Peninsula’s flora of vascular plants (Chin 1977). About 20% of these species are restricted to limestone substrates. The great majority of these karst limestone hills lie outside the network of Totally Protected Areas that include National and State Parks, Wildlife Sanctuaries and Permanent Reserved Forests, so are particularly vulnerable to disturbance from mining, fire, ecotourism and cave temples. Nationally, limestone hills are classified as Environmentally Sensitive Areas.

Both the new species described below are each known from a single karst limestone hill. *Coleuskunstleri* is also restricted to karst limestone but is known from two widely separated localities. All three are endemic in Peninsular Malaysia. For assessing conservation status, the IUCN criteria and categories (IUCN Standards and Petitions Committee 2019) are followed. Extent of Occupancy (EOO) for species restricted to limestone habitats is assumed to be the same as its Area of Occupancy (AOO) due to substrate restriction. *Coleushairulii*, *C.kunstleri* and *C.rafidahiae* are assessed as Critically Endangered because the EOO and AOO are below 100 km^2^ and 10 km^2^ respectively and none lies within a Totally Protected Area, which means that they are vulnerable to habitat disturbance or loss. In addition, *Coleushairulii* and *C.rafidahiae* are each known from a single locality, while *C.kunstleri*, although known from two localities (Kuala Dipang, Perak and Pulau Langgun, Langkawai, Kedah) is unlikely to still grow in Kuala Dipang because it has been, and is, heavily disturbed by mining and, although the area has been regularly visited for botanical collecting, this species has not been recollected for more than 130 years. Today, it is therefore extant at a single locality. In contrast, *C.scutellarioides* does not grow on limestone and is widespread, though not common, on the fringes of lowland forest. It is not endemic. For endemic species, the conservation status is the global conservation status, while for *C.scutellarioides*, it is the regional conservation status that applies only to the Peninsular Malaysian population (Chua and Saw 2006).

Botanical exploration has shown that many species (about 15%) of the limestone flora have extremely local distributions (Kiew et al. 2017). One hundred taxa (species, subspecies or varieties) are known from a single karst hill, while a further 92 are known from two to four karst hills or islands. These species are particularly endangered by extinction. The three *Coleus* species that are endemic and restricted to limestone are further examples of this phenomenon.

## ﻿Taxa cultivated in Peninsular Malaysia

Coleus, *Coleusscutellarioides*, is widely available in nurseries and popular as an ornamental plant for its incredible variety of leaf colour (reds, purples and yellows) and patterns of variegation. In Peninsular Malaysia, it seldom flowers. Its local Malay name is *pokok ati-ati.* Several minor medicinal uses have been ascribed to it (see below under species).

Indian borage, *Coleusamboinicus* Lour., said to be native to India, is commonly grown in pots in home gardens for its medicinal value (for a dry cough a decoction of the leaves is drunk to sooth the throat). It rarely flowers and does not fruit. It is readily distinguished from the other species by its thick, hairy, succulent, harshly aromatic leaves. Its local Malay name is *daun bangun-bangun*.

*Plectranthus* ‘Mona Lavender’ is currently a very popular floriferous species with deep purple flowers that grows well in the highlands at Cameron Highlands, Pahang, and has caused confusion with the public because it is marketed as a true lavender (Kiew 2016). It is a hybrid developed in the 1990s in the Kirstenbosch Botanic Gardens, Cape Town, South Africa.

The edible *C.rotundifolius* (Poir.) A.Cheval & Perrot, although once reported from Peninsular Malaysia (Burkill 1966), is today not planted even as a curiosity (Kiew 2016).

## ﻿Excluded species

Suddee et al. (2004) reported *Plectranthusglabratus* (Benth.) Alston (now known as *Coleuspaniculatus* Benth.) from Penang, Peninsular Malaysia, based on a single specimen, *Haniff & Nur 4020* (BM, K). However, examination of the specimen label shows that it was in fact collected from ‘Rilau Seng Penong, Lower Siam’. At the time of collection (12 December 1918) Haniff and Nur were collecting plants from Pulau Panji and Pulau Pungah in southern Thailand. This species is therefore excluded from the Peninsular Malaysian flora.

## ﻿Taxonomy

### ﻿Key to *Coleus* species in Peninsular Malaysia

**Table:** 

1	Inflorescences spike-like with distinct condensed cymes	**2**
–	Inflorescence thyrsoid, flowers in lax cymes, single or in 2–3s	**3**
2	Inflorescences (14–)20–38 cm long. Lower median lobes of the fruiting calyx fused, obovate with two apiculate teeth	**3. *C.monostachyus^1^***
–	Inflorescences 5–10(–25) cm long. Lower, median lobes of the fruiting calyx fused, spathulate and deeply bifid	**5. *C.scutellarioides***
3	Lamina 2–3 times longer than wide. Flowers yellowish white	**4. *C.rafidahiae***
–	Lamina less than twice as long as wide. Flowers purple or greenish white	**4**
4	Leaves 5–6 × 3.3–4.5 cm. Branches from the inflorescence axis 3–5 times shorter than the 13–18 cm-long inflorescence. Cymes lax with 6–8 flowers, flowers ca. 20 mm long, lower lip deep purple	**1. *C.hairulii***
–	Leaves 6–12 × 4–6 cm. Branches from the inflorescence axis 2–3 times shorter than the 5–6 cm-long inflorescence. Cymes single-flowered, c. 10 mm long, greenish white or pale pinkish	**2. *C.kunstleri***

### ﻿Species

#### ﻿1. Coleus hairulii

Taxon classificationPlantaeLamialesLamiaceae

Kiew sp. nov.

B8A5CB9B-55ED-51B8-BBBF-77469751CFA9

urn:lsid:ipni.org:names:77234076-1

##### Diagnosis.

Most similar to *Coleuspaniculatus* Benth. (as *Plectranthusglabratus* (Benth.) Alston in Suddee et al. 2004) from southern Thailand in its habit (both are low, little branched or unbranched herbs), in their inflorescences with conspicuous side branches and cymules with more than 3 flowers, but *Coleushairulii* (Figure 2) is different (Table 1) in its short branches from the inflorescence axis 2.5–4 cm long, i.e. 3–5 times shorter than the inflorescence length (vs. branches from the inflorescence axis 5.5–10 cm long and 2–3 times shorter than the inflorescence), and its calyx 6–7 mm long in fruit (vs. 4–6 mm long).

**Table 1.** *Coleushairulii* and *C.paniculatus* compared.

Character	*C.hairulii*	*C.paniculatus*
Stem		
*height* (*cm*)	15–30(–40)	to 50
*diameter* (*mm*)	ca. 2	3–4
Leaves		
*petiole length* (*cm*)	2–6.5	4–7
*lamina length × width* (*cm*)	5–6 × 3.3–4.5	1.5–12 × 1–7.5
*margin*	shallowly crenate	regularly crenate
*vein no.*	4–5	ca. 6
Inflorescences		
*total length* (*cm*)	13–18	17–30
*length side branches* (*cm*)	2.5–4	5.5–10
Flowers		
*calyx length in fruit* (*mm*)	6–7	4–6
*length anterior corolla lobe* (*mm*)	9–14	5–8
*inner surface of anterior lobe*	densely hirsute	pubescent

##### Type.

Malaysia. Peninsular Malaysia: Kedah, Baling District, Gunung Pulai, Kepala Gajah, trail to summit 5°40.05'N, 100°53.03'E, 19 Nov 2019, Wan Syafiq & R. Kiew FRI 99402 (holotype KEP!; isotypes K!, SAN!, SAR!, SING!).

##### Description.

Soft unbranched herb, 15–30(–40) cm tall, erect becoming decumbent, not aromatic, stem quadrangular, ca. 2 mm thick, fleshy, glabrous. ***Leaves*** glabrous; petiole slender, 2–6.5 cm long, decreasing in length in the upper leaves; lamina thinly membranous, ovate to broadly ovate, 5–6 × 3.3–4.5 cm, base rounded to truncate, margin shallowly crenate, apex acute to acuminate, pale green beneath; veins 4–5 on either side of midrib. ***Inflorescence*** thyrsoid, terminal, erect, 13–18 cm long, shortly glandular pubescent, rachis and branches pale reddish, peduncle 7–9.5 cm long, 3–4 tiers of short side branches from the inflorescence axis, 2.5–4 cm long; cymes single-flowered; bracts sessile, foliose, lanceolate, apex acute, 6–7 × 2–3 mm; pedicels attached slightly above centre of calyx, 2.5–4 mm long at anthesis, 4–5 mm in fruit. ***Flower*** pendent; *calyx* obliquely campanulate, reddish brown, posterior lip rosy red, 2–3 mm long at anthesis, 10-veined, unequally 5-lobed, minutely puberulent, in fruit 6–7 mm long, 2.5–3 mm wide, posterior lip spathulate, apex subacute, 2–2.5 × 3 mm, anterior lip with 2 lateral and lower lobes sharply acute, slightly upcurved, lateral lobes ca. 2–2.5 mm long, slightly longer than the lower 2.5 mm; *corolla* (9–)20 mm long, minutely puberulent outside, pale or bluish purple except for the white posterior lip, tube sigmoid, basal ca. 3 mm very narrow ca. 1 mm wide, abruptly decurved and dilating to 3–4 mm wide across the mouth, posterior lip erect, ca. 8 × 6 mm, almost rounded with a broad median lobe deeply notched at apex, lateral lobes narrow, slightly longer than the median lobe, anterior lip boat-shaped and enclosing the stamens and style, 9–14 mm long, 5–6 mm deep and wide with a dense tuft of long translucent hairs in the inner lower half; *stamens* hardly exserted, only anther tips visible, in 2 pairs, filaments 7 and 8 mm long, anthers slightly obovoid, ca. 0. 5 mm long; stigma slightly bilobed, slightly exserted from tip of lower corolla lip. ***Nutlets*** orbicular in outline, bilaterally flattened, ca. 1 mm long; testa reddish brown, smooth.

##### Distribution.

Endemic in Peninsular Malaysia, Kedah, Gunung Pulai, known only from two populations (trail to Gunung Pulai and Kampung Sungai Limau) at a single locality (Map 1).

##### Provisional conservation status.

Critically Endangered B1ab(i,iii,iv). The species is known only from one locality, with a low population size, estimated at less than a hundred individuals. The hill is threatened by a proposal for a cement quarry. (Assessed by A.R. Rafidah).

##### Ecology.

Restricted to a limestone karst hill at ca. 90–155 m on lower slopes of the hill, growing in soil-filled cracks in the limestone rocks on or near wet, shaded vertical cliffs or between deep pinnacles on a low summit, locally common in a restricted area. It is not known if it is short-lived or an annual plant.

##### Etymology.

**Figure 1.**
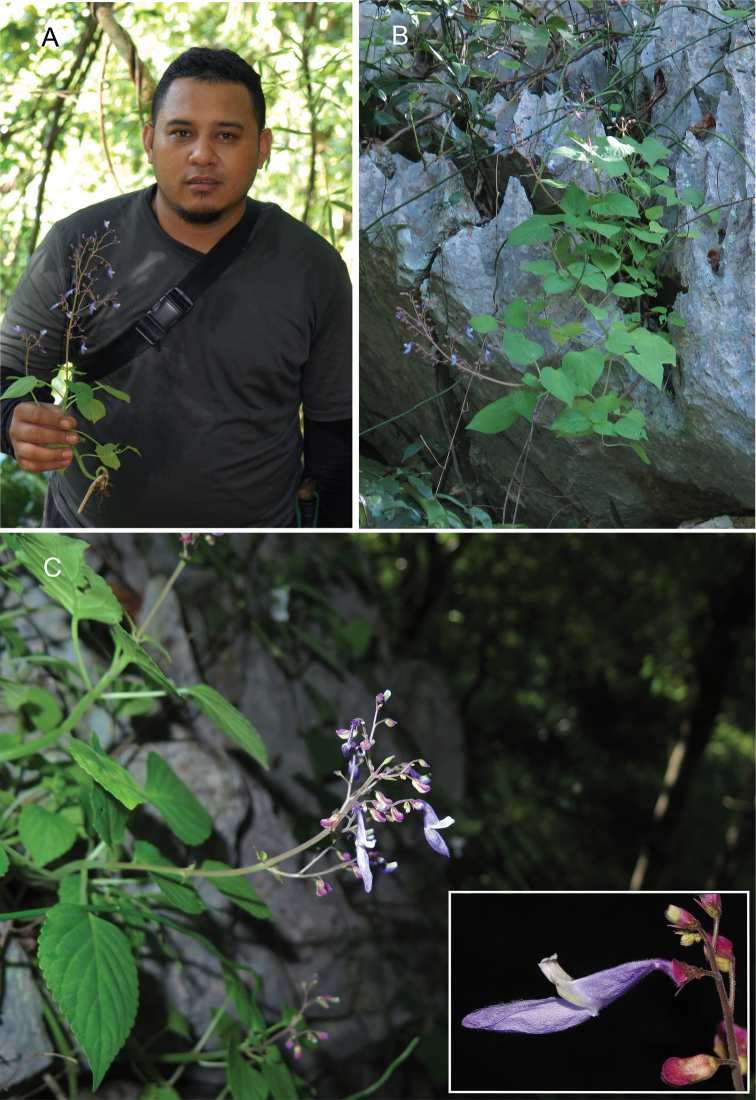
*Coleushairulii* Kiew, sp. nov. **A** Mohd. Hairul bin Mohd. Amin with a flowering specimen **B** habit and limestone habitat **C** inflorescence and flower enlarged. (Photographs: **A** by AR Ummul-Nazrah **B** by Wan Mohamad Syafiq bin Wan Putra and **C** by AR Rafidah).

Named for Mohd. Hairul b. Mohd. Amin (b. 1984), field staff in the KEP Herbarium, Forest Research Institute Malaysia, who first collected this species (Figure 1).

**Figure 2.**
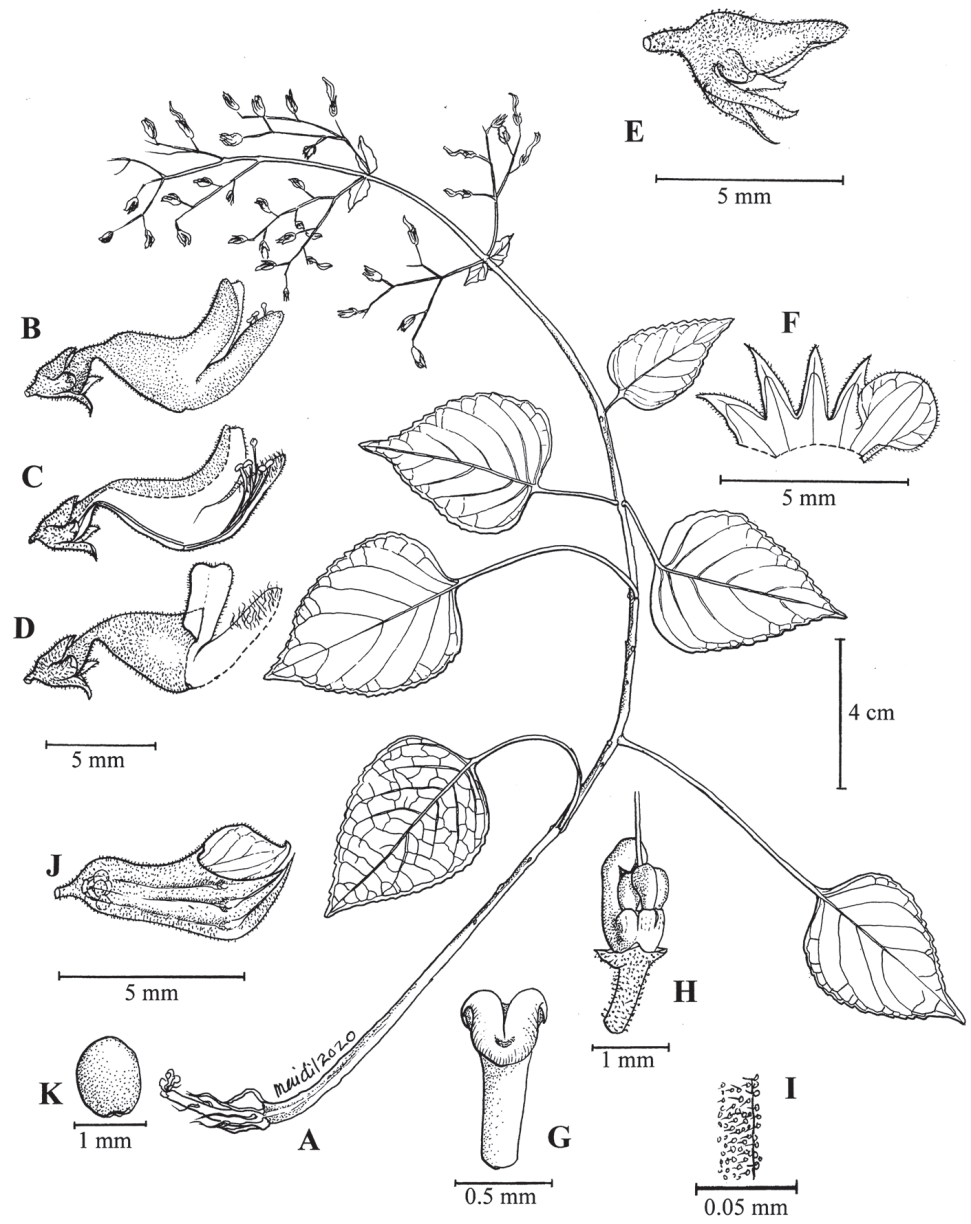
*Coleushairulii* Kiew **A** flowering plant **B** flower side view **C** flower side view with corolla cut away to show stamens **D** flower side view to show hirsute patch on anterior lip **E** calyx in flower **F** calyx opened **G** stamen apex **H** ovary side view **I** glandular hairs of the pedicel **J** calyx in fruit **K** seed. (All from *FRI 94402*. Drawn by N. Mohamad Aidil).

##### Additional specimen examined.

**Peninsular Malaysia, Kedah**: Baling Hill, Kampung Sungai Limau, 5°40.38'N, 100°53.23'E, 17 Nov 2011, Mohd. Hairul et al. FRI 54055 (K!, KEP!, SING!).

##### Note.

In discussions with AJ Paton (*pers. comm*.), he pointed out that the type of *Plectranthusglabratus* (Benth.) Alston (now *C.paniculatus*) from Chennai (Madras) is a more robust plant with a thicker stem, bigger leaves and relatively shorter branches of the inflorescence compared with the three specimens from southern Thailand. *Coleushairulii* resembles specimens from southern Thailand that are also all from limestone rocks (Suddee et al. 2004).

#### ﻿2. Coleus kunstleri

Taxon classificationPlantaeLamialesLamiaceae

(Prain) A.J. Paton in Paton et al., Phytokeys 129 (2019) 63.

8CA3B4DA-B04F-5703-A7A2-893C9418F14F

Homotypic synonym: Plectranthuskunstleri Prain, J. Asiat. Soc. Bengal 74, 2: 706 (1907); Ridley, Fl. Mal. Penin. 2: 646 (1923); Keng, Gard. Bull. Singapore 24: 151 (1969), Fl. Malesiana Ser. I, 8: 392 (1978); Chin, Gard. Bull. Singapore 32: 160 (1979); Turner, Gard. Bull. Singapore. 47: 272 (1996 ‘1995’); Bramley, Fl. Malesiana Ser. I, 23: 293 (2019).Type: Malaysia. Peninsular Malaysia: Perak, Kuala Dipang, top of limestone hills, 130–200 m. Sept. 1885. *Kunstler* (*King’s collector*) *8240* (holotype CAL; isotypes BM!, K!).

##### Description.

Erect herb, almost shrubby, 0.5–1.2(–1.5) m high. ***Stem*** and branches finely puberulous, angular. ***Leaves*** with petiole 1–4.5 cm; lamina membranaceous, ovate to broadly ovate, 6–10(–13) × 4–6 cm, base truncate or shortly cuneate, margin elsewhere remotely crenate, apex acute, very sparsely pubescent on the main and secondary veins on both surfaces, sometimes with a whitish line down midrib. ***Inflorescence*** terminal, paniculate, 10–20 cm long, the lower branches of the inflorescence axis 5–6 cm, decreasing in length towards apex. Bracts ovate, acute, 2–3 mm long, caducous. Pedicels 3–4 mm long, glandular-puberulent. ***Flowers*** spreading, (not pendent); *calyx* obliquely campanulate, 1–2(–2.5) mm long, in fruit 5–7 mm, shortly hirsute and sparingly gland-dotted, unequally 5-lobed; upper lip ovate, broadly ovate or sub-rounded, lower lip with two lateral ovate-acute lobes shorter (about ¾) than the lower central lobes but later almost equal in fruit, the two central lobes subulate, connate beneath; *corolla* waxy white or greenish white (*Kunstler 8240*) or purplish pink (*Chin 1760*), 7–8(–10) mm long, puberulent, tube sigmoid, decurved, only slightly gibbous near the base, posterior lip short, erect, anterior lip concave, boat-shaped, c. 4 mm long; *stamens* not exserted, lying within the anterior lip, free above point of insertion on the corolla tube. ***Nutlets*** oblong ovoid, black, *ca.* 1 mm long.

##### Distribution.

Endemic in Peninsular Malaysia, Perak – Kinta (no specific locality) and Kuala Dipang; and Kedah – Langkawi, Pulau Langgun (Map 1).

##### Provisional conservation status.

Critically Endangered B1ab(i,iii). Limestone hills in Perak are regularly visited for botanical collecting but this species has not been recollected there for over a hundred years and the majority of karst limestone hills in the Kinta Valley are heavily disturbed by quarries. The species was last collected in the 1970s from Pulau Langgun. (Assessed by A.R. Rafidah).

##### Ecology.

Restricted to limestone rocks, in shade, at 50–200 m elevation, either in open habitats on top of a limestone hill to 200 m (*Kunstler 8240*) or on huge boulders rooting in rock crevices and on humus accumulated on boulders under shade of scattered trees at ca. 50 m (*Chin 1760*).

##### Etymology.

H.H. Kunstler, a German botanist, who collected plants in 1880–1886 mostly from Perak, Peninsular Malaysia, for Sir George King, Director of Calcutta Botanic Garden. He is also recorded on herbarium labels as ‘King’s Collector’.

##### Additional specimens examined.

**Peninsular Malaysia.** Perak: Kinta (*Kunstler 7143*, BM!). Kedah: Langkawi, Pulau Langgun (*Chin 1760*, KLU!).

#### ﻿3. Coleus monostachyus

Taxon classificationPlantaeLamialesLamiaceae

(P.Beauv.) A.J.Paton, Phytokeys 129 (2019): 76.

8AD3FED0-7B31-5EFE-B736-AA187CEA87DB

Homotypic synonym: Plectranthusmonostachyus (P.Beauv.) B.J. Pollard, Kew Bull. 56: 980 (2001); Solenostemonmonostachyus (P.Beauv.) Briq. *in* H.G.A.Engler & K.A.E.Prantl, Nat. Pflanzenfam. 4(3a): 359. 1897. Basionym: Ocimummonostachyum P.Beauv., Fl. Oware 2: 60, t. 95 (1818). Type: Africa, Benin, *Palisot de Beauvois s.n.* (holotype G, *n.v*.).
Subsp. monostachyus. Chung et al., Nature in Singapore 8: 1 (2015); Kiew, Conservation Malaysia 23: 6 (2016).

##### Note.

The description is based on specimens from Peninsular Malaysia.

##### Description.

Erect herb, almost shrubby, not aromatic, without tubers. ***Stem*** quadrangular, densely hairy on the angles, hairs descending ca. 0. 5 mm long, green, woody but brittle, to 30 cm tall, ca. 4 mm diameter, at first unbranched, flowering at ca. 20 cm tall, soon branching near the base, internodes (2.5–)4.5–6.5 cm, branches ascending, to 29 cm long. ***Leaves*** held horizontally; petiole 2–5.6 cm long, narrowly winged in the upper third, grooved above, densely hairy on the angles; lamina broadly ovate, (3.5–)6–13.5 × (2.5–)4.5–11.5 cm, base rounded or slightly truncate, shortly decurrent into the petiole, margin shallowly crenate, apex acute, tip rounded, membranous, glabrous and completely dull green above, pale beneath, lateral veins ca. 4 on either side of the midrib, impressed above, beneath prominent and finely short hairy. ***Inflorescence*** terminal on stem and branches, spicate, ca. 14 cm long in unbranched plants, in branched plants 21–38 cm, often with a subsidiary pairs at the base ca. 16.5–23 cm long, peduncle short 3–5 cm, 8–8.5 cm in inflorescences from the lower branches; peduncle and rachis quadrangular, finely pubescent on the angles; verticils 1–2 cm apart at the base, ca. 0.5 cm apart near the apex, each verticil with two sessile, condensed cymes each with 3–5 flowers. Bracts broadly ovate apex caudate, keeled, ca. 4 × 2.5 mm, pale green, deflexed and appressed to rachis. Pedicels reddish on the upper side, slightly excentrically attached behind the posterior lip, minutely pubescent, 1.5–2 mm long. ***Flower*** with *calyx* funnel-shaped, pale green, densely pubescence 2–3.5 mm long, in fruit 5–5.5 mm long, upper lip curved upwards, oval, minutely punctate at the apex, ca. 4–4.5 mm long, lower lip broadly oval with two fine apical teeth, curved upwards and closing the throat; ***corolla*** 8.5–10 mm long, minutely pubescent outside, tube abruptly decurved above the calyx, dilating to the mouth, white except for the upper lip and lateral lobes outlined in deep purple and the deep purple lower lip 3–4 mm long; *stamens* fused at base, filament white, glabrous, anthers, ca. 0.25–0.3 mm long, deep purple; *stigma* ca. 0.4 mm long, positioned above the anthers. ***Nutlets*** 4, plain brown, broadly ovoid, almost 1 mm long, producing mucilage when wet.

##### Distribution.

Native in tropical West Africa, this is a recent introduction into Peninsular Malaysia and Singapore, probably an escape from the horticultural trade (Kiew 2016). First collected in Peninsular Malaysia in 2003, it is now naturalised and since about 2010 has rapidly become widespread. Apparently, it has not yet been recorded from Southeast Asia (Suddee et al. 2004).

##### Ecology.

In Peninsular Malaysia, it is found in light shade to fully exposed conditions in almost all lowland habitats associated with disturbance, e.g. roadsides, plantations, farms and gardens. It begins to flower at 20 cm tall, and its many-flowered spikes produce fruits that shatter at the slightest touch, scattering hundreds of seeds. This makes it a weed that is extremely difficult to eradicate. The seeds are sticky and may be dispersed by animals or water but long distance dispersal is probably effected by soil on vehicles or in planting material. It therefore threatens to become a noxious weed in nurseries and gardens where it cannot be exterminated by herbicides.

##### Etymology.

Latin, *mono*- = one or single; *stachys* = ear of corn or spike, referring to the inflorescence.

##### Additional Peninsular Malaysian specimens examined.

**Johor**: Senterre et al. s.n. 30 Sept 2003 (KEP!). **Kelantan**: Felda Chiku, Nazrul et al. FRI 83177 31 August 2015 (KEP!). **Selangor**: Bukit Nanas, Norzielawati et al. FRI 83050 (KEP!), Kepong, Forest Research Institute Malaysia, Kiew FRI 81947 13 Jan 2016 (KEP!); Rasa, Kiew FRI 655542 17 April 2010 (KEP!); Subang Rafidah et al., FRI 75694 4 April 2013 (KEP!).

#### ﻿4. Coleus rafidahiae

Taxon classificationPlantaeLamialesLamiaceae

Kiew sp. nov.

B004B890-F1C8-5AA4-8E74-D7FF37170965

urn:lsid:ipni.org:names:77234077-1

##### Diagnosis.

Unusual among Malaysian *Coleus* species in its branched inflorescence with yellowish white flowers (Figure 5). It resembles *C.calcicola* Murata from Thailand in its branched thyrsoid inflorescence with 1-flowered spaced cymes, in its calyx and yellowish white corolla but it is different in its larger laminas, 8.5–13.5 × 4–5.5 cm with longer petioles 2.5–8 cm long (vs. laminas 3–8 × 1.5–3 cm with petioles 0.5–1.5 cm long in *C.calcicola*).

**Figure 3.**
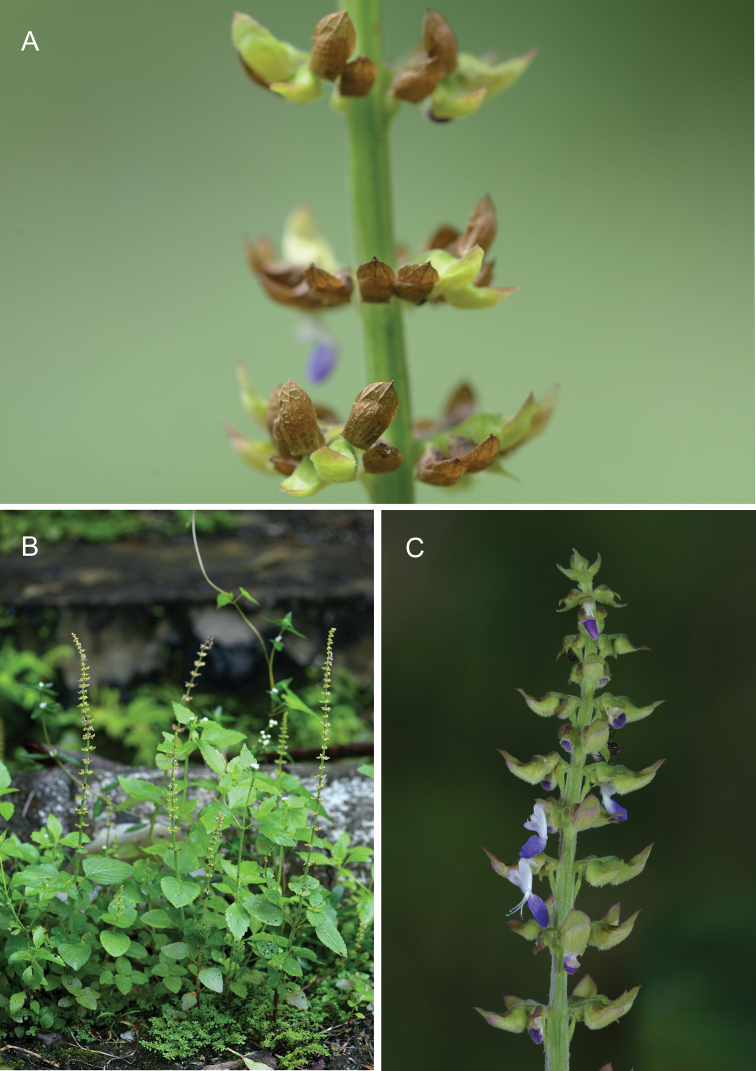
Coleusmonostachyus (P.Beauv.) A.J.Paton subsp. monostachyus**A** portion of fruiting spike **B** habit **C** top of flowering spike. (Photographs by PT Ong).

**Figure 4.**
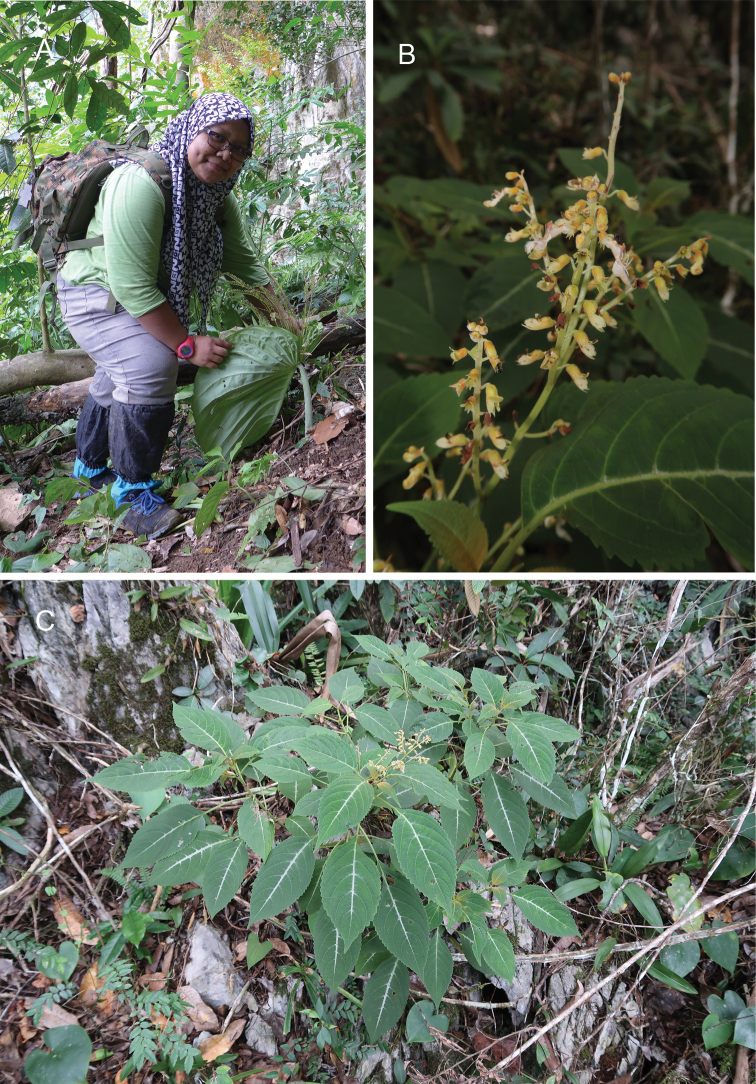
*Coleusrafidahiae* Kiew, sp. nov. **A** AR Rafidah on limestone hill with a *Monophyllaea***B** inflorescence **C** flowering plant.

**Figure 5.**
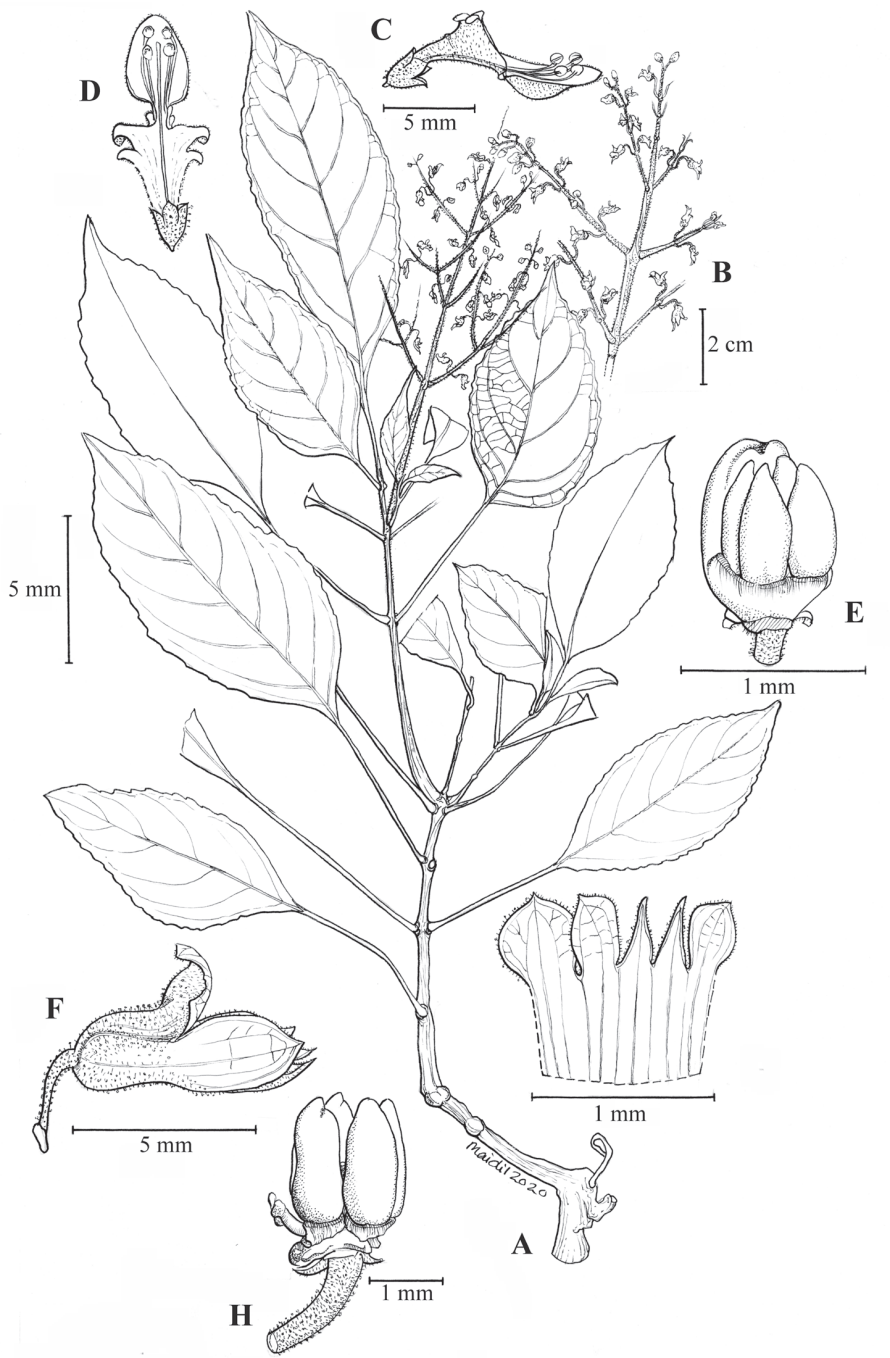
*Coleusrafidahiae* Kiew **A** flowering plant **B** portion of inflorescence **C** flower side view **D** flower from above **E** ovary **F** calyx in fruit **G** calyx opened **H** seeds side view. (All from *FRI 87271*. Drawn by N. Mohamad Aidil).

##### Type.

Peninsular Malaysia. Kelantan: Kuala Betis, Gunung Biol, 4°54.01'N, 101°45.33'E, 19 July 2017, Imin, Ong & Wan Shafiq FRI 87271 (holotype KEP!; isotype K!).

##### Description.

Erect perennial, rather shrubby herb, to ca. 1 m tall. ***Stems*** brown and woody, branched, quadrangular, internodes 2–3 cm apart, ca. 4 mm thick, glabrous. ***Leaves***: petiole slender, (2.5–)5.5–8 cm long, glabrous; lamina broadly elliptic to slightly ovate, 8.5–13.5 × 4–5.5 cm, base cuneate to truncate, margin crenate, apex acuminate, acumen 0.5–1.5 cm long, puberulous and densely dotted with minute glands on both sides; midrib white; veins inconspicuous beneath, 6–7 pairs on either side of midrib, midrib and veins slightly impressed above, slightly prominent beneath. ***Inflorescence*** terminal, branched lax thyrse, to 19 cm long, peduncle ca. 3.5 cm long, branches 5–6 cm long, 1.2–2.5 cm apart, glabrescent with a few minute hairs persisting near the apex, cymes 1-flowered, spaced on the branches. Bracts sessile, ovate, apex acute, 2–3 mm long, ciliate, pubescent, early caducous. Pedicels pubescent with gland-tipped hairs, ca. 2 mm long at anthesis, 3–4 mm in fruit. ***Flower*** with *calyx* campanulate, yellowish, pubescent with gland-tipped hairs, ca. 3.5 mm long at anthesis, in fruit 6–8 mm long, posterior lip erect, ovate, shortly acuminate at apex, constricted at base, 2–2.5 × 3–3.5 mm, margin entire; anterior lip 3.5–4 × 2–2.5 mm deeply divided into two teeth equal in length, ca. 2 mm long, slightly longer than posterior lip, lateral teeth obovate, 2–3 × 2–3 mm, spreading; tube 10-veined; *corolla* yellowish white, 7–12 mm long, minutely pubescent; posterior lobes minute rounded at apex; anterior lip ovate-oblong, 3–5 mm long, concave, shortly pubescent inside; tube ca. 7 mm long, not abruptly decurved above the calyx, dilated towards the throat; *stamens* included in anterior corolla lip; *style* bifid, subequal to anterior stamens; nectary exceeding ovary. ***Nutlets*** brown, ellipsoid, 1.75 mm long, 1.25 mm wide, smooth.

##### Distribution.

Endemic in Peninsular Malaysia, Kelantan: Kuala Betis, Gunung Biol. Known only from two specimens from the type locality (Map 1).

##### Provisional conservation status.

Critically Endangered B1ab(i,iii,iv). The species occurs on a single karst limestone hill that lies outside the network of Totally Protected Areas. It is known from a single small population. (Assessed by A.R. Rafidah).

##### Ecology.

Near the summit of a karst limestone hill at 325 m altitude.

##### Etymology.

Rafidah binte Abdul Rahman (b. 1981), KEP botanist, active contributor to revisions for the Flora of Peninsular Malaysia and the team leader of the project ‘Towards a Conservation Strategy/Policy for Limestone Hills in Peninsular Malaysia: Understanding and Documenting Plant Biodiversity with Focus on Kelantan and Perlis Limestone Hills’ funded by the National Conservation Trust Fund (Figure 4).

#### ﻿5. Coleus scutellarioides

Taxon classificationPlantaeLamialesLamiaceae

(L.) Benth.

10D126E5-0F2E-5F0E-AB2D-9DDB104E1929

*in* Wall., Pl. Asiat. Rar. 2: 15 (1830); Keng, Gard. Bull. Singapore 24: 51 (1969). Coleusscutellarioidesvar.scutellarioides Keng, Gard. Bull. Singapore 24: 53 (1969). Basionym: Ocimumscutellarioides L., Sp. Pl. ed. 2, 2: 834 (1763) (as Ocymum). Type: Majana (alba et rubra) *in* Rumphius, Herb. Amboin. 5: 291, t. 101 (1747), (lectotype, designated by Merrill, Interpr. Rumph. Herb. Amboin.: 460 (1917).) Homotypic synonyms: Plectranthusscutellarioides (L.) R.Br., Prodr. Fl. Nov. Holl.: 506 (1810); Blume, Bijdr. Fl. Ned. Ind.: 837 (1826); Keng, Fl. Malesiana Ser. I, 8: 389 (1978); Suddee et al., Kew Bull. 59: 410 (2004); Kiew in Henderson’s Malaysian Wild Flowers Dicotyledons: 298 (2014); Bramley, Fl. Malesiana Ser. I, 23: 296 (2019). Solenostemonscutellarioides (L.) Codd, Bothalia 11: 439 (1975); Turner, Gard. Bull. Singapore 47: 273 (1996 ‘1995’). Heterotypic synonyms: Coleusatropurpureus Benth. *in* Wall., Pl. Asiat. Rar. 2: 16 (1830); Prain, J. Asiat. Soc. Beng. 74, 2: 706 (1907); Ridley, Fl. Mal. Penins. 2: 646 (1923). Syntypes: Singapore *Wall. Cat. 2733A* (syntypes K!, K-W!). Coleusblumei Benth., Labiat. Gen. Sp.: 56 (1832). Type: Java, *Blume s.n.* (lectotype L, designated by Suddee et al., Kew Bull. 59: 410 (2004).)

##### Note.

Here are included only references that cite Malaysian specimens. For synonomy under *Coleus* see Paton *et al*. Phytokeys 129 (2019) 1–158, for *Plectanthus* for continental South East Asia see Suddee et al. (2004) and for Malesia see Bramley (2019).

The description provided here refers only to wild plants from Peninsular Malaysia.

##### Description.

Erect or ascending sparsely branched herb, 30–60 cm tall, aromatic, without tubers. ***Stem*** and branches finely pubescent to glabrous. ***Leaves*** with petiole 1–5 cm; lamina ovate, (2.5–)4–7.5 × (2–)3–4 cm, plain dark green, base rounded or sometimes truncate, margin crenate with rounded teeth, apex acute, tip rounded, upper surface glabrous or with short hairs, lower surface pubescent on the main and secondary veins. ***Inflorescences*** terminal, unbranched spike or sometimes branched at base, to 40 cm long, flowers in few- to many-flowered verticils or in irregularly branched cymes, peduncles of the lateral cymes short or elongated. Bracts narrowly ovate to ovate, apex acute, pubescent, 2–8 mm long, caducous. Pedicels 1–2 mm long in flower, extending in fruit. ***Flower***: *calyx* campanulate, 2–3 mm long, in fruit 4–8 mm, minutely pubescent; posterior lobe broadly ovate, subacute, recurved and erect, two lateral lobes of anterior lip short, oblong-obtuse, truncate or rounded, rarely mucronate with a tiny apiculate apex, about half as long as central lobes of anterior lip, these oblong to subulate, connate for two thirds their length, acuminate at apex, longer than posterior lobe, anterior lobe divided into 2 points; *corolla* 8(–13)–16 mm long, tube abruptly decurved above the calyx, white, 4–5, ca. 7.5 mm long, dilating widely to the throat, with scattered hairs; posterior lip with lobes pubescent, the two central lobes rounded, anterior lip deep or rosy violet, 4–5(–9) mm long; *stamens* not exserted from anterior lip. ***Nutlets*** broadly ovate or orbicular, brown to black, shining, 1–1.2 mm long, minutely tuberculate, mucilaginous when wet.

##### Note.

Ridley (1923) and Burkill (1966) considered the coleus of gardens (i.e. *Coleusblumei*) was introduced from Java and was distinct from Malayan *C.atropurpurea*. These two species are recognized as conspecific and are currently known as *Coleusscutellarioides* (Paton et al. (2019) with the result that the description has expanded to include the huge range in leaf size, shape and colour of the ornamental forms that are not seen in the wild form of Malaysian population. The description above is based on the wild form that has smaller, plain green leaves. Unlike *Scutellariadiscolor* Wall. ex Benth., it is not found deep in forest and is seldom found in flower. That it is usually associated with forest edge, often close to habitation, suggests that many of these populations have been established for their medicinal properties.

On account of it being extremely variable, Keng (1978) and Bramley (2019) did not recognise subspecific taxa, there being too many intermediate specimens. A view that is followed here.

##### Distribution.

SE Asia (India, Myanmar, Thailand, Indo-China, S. China, Taiwan, throughout Malesia to Australia, Melanesia (Solomon Is.) and Polynesia. In Peninsular Malaysia, widespread (Johor, Melaka, Penang, Perak, Pahang and Selangor).

##### Provisional conservation status.

Least Concern. (Assessed by A.R. Rafidah).

##### Ecology.

In Peninsular Malaysia, from forest edge and often near villages throughout the lowlands to 350 m elevation (Kiew 2014), sometimes in forest by streams but not from limestone hills as reported by Keng (1978). That the forest localities are often close to villages and it has medicinal uses suggests these populations may have been planted, particularly because this species seldom flowers but can easily be propagated by stem cuttings.

##### Etymology.

Greek -*oides* = resembling; *Scutellaria* L., a genus in Lamiaceae.

##### Uses.

In Peninsular Malaysia, it has always been commonly known as ‘coleus’ and in Malay as *pokok ati-ati*. Cultivated forms are popular ornamental plants and come in a great variety of multicoloured, variegated foliage. In Cameron Highlands, they are grown in great quantities for home gardens. The colour of plants grown in the highlands, ca. 2000 m, is more vivid than those grown in the lowlands.

It is a minor medicinal plant used to cure a wide range of ailments. Burkill (1966, pg. 643) reported it was a remedy for heart disease, heart burn, inflammation of heart; sensitive skin, stimulates digestion and for congestion of the liver that causes swellings of the hands and feet, amongst other ailments. Among the aboriginal population, the Besis people (now more commonly known as the Mah Meri) call it *torek*, and plant it around their graves. They also traditionally use it as the brush used for sprinkling holy rice-gruel over a new clearing (Burkill 1966).

##### Peninsular Malaysia specimens examined

(* indicates specimens collected in villages that are presumed to be cultivated): **Johor**: Pulau Aor *Fielding s.n. 1892*. **Melaka**: *Griffith 39*57* 1845 (K), Burkill 35515*. **Pahang**: Bentong *Burkill & Md Haniff SFN 16536*; Pekan *Burkill & Md Haniff SFN 17728*; Sungai Telom *Poore 538* roadside; Tapah *Burkill & Md Haniff SFN 13527*. **Penang**: Wallich s.n.* 1829 (K); *Curtis 466* Paya Tobong 1892; Sinclair SFN 39391* village, Pulau Betong. **Perak**: Bagan Datuk *Md Haniff SFN 16273*; Batu Kurau *Md Haniff & Sa’at SFN 13272*; Gerik *Burkill & Md Haniff SFN 13647*; Kampung Gajah *Md Haniff SFN 16299*; Kota Setir *Md Haniff SFN 15924*; Kuala Kangsar to Kota Lama, *Md Haniff SFN 15562*; Lubuk Merbok, *Md Haniff SFN 15985*, *Md Haniff SFN 16004*; Tanjung Malim – *Burkill & Md Haniff SFN 13500*; Telok Anson *Burkill & Md Haniff SFN 15945*, *Md Haniff SFN 10315.***Selangor**: Ulu Gombak *T & B 2823* roadside [leaves multicoloured], *Md Nur SFN 3423*2 by stream; Kuala Lumpur Ridley s.n.* 1890 bunga ati-ati merah. **Terengganu**: Bundi *Rostado s.n.*

## Supplementary Material

XML Treatment for Coleus hairulii

XML Treatment for Coleus kunstleri

XML Treatment for Coleus monostachyus

XML Treatment for Coleus rafidahiae

XML Treatment for Coleus scutellarioides

## Footnotes

Note. In life, Coleus monostachyus is immediately recognised by its erect inflorescences (Figure 3). It begins to flower when just 15 cm tall. In contrast, C. scutellarioides in Peninsular Malaysia seldom flowers even in cultivation. Its inflorescences too are shorter. Coleus scutellarioides is conspicuous in being the only Malaysian species with coloured leaves. Vegetatively, it is extremely variable due to the myriad of selections for leaf shape, size and colour. It is for this reason that the key uses inflorescence and calyx characters.
